# Paraoxonase 3: Structure and Its Role in Pathophysiology of Coronary Artery Disease

**DOI:** 10.3390/biom9120817

**Published:** 2019-12-03

**Authors:** Kumari Priyanka, Surjit Singh, Kirandip Gill

**Affiliations:** 1 Department of Biochemistry, Postgraduate Institute of Medical Education and Research, Chandigarh 160012, India; Kdgill2002@yahoo.co.in; 2 Departments of Immunopathology, Postgraduate Institute of Medical Education and Research, Chandigarh 160012, India; 3 Department of Internal Medicine, Postgraduate Institute of Medical Education and Research, Chandigarh 160012, India

**Keywords:** Paraoxonase 3, polymorphisms, PON3 activity, statinase, coronary artery disease, HDL

## Abstract

Spanning three decades in research, Paraoxonases (PON1) carried potential of dealing with neurotoxicity of organophosphates entering the circulation and preventing cholinergic crisis. In the past few years, the Paraoxonase multigene family (*PON1, PON2, PON3*) has been shown to play an important role in pathogenesis of cardiovascular disorders including coronary artery disease (CAD). The *PON* genes are clustered in tandem on the long arm of human chromosome 7 (q21, 22). All of them have been shown to act as antioxidants. Of them, PON3 is the least studied member as its exact physiological substrate is still not clear. This has further led to limitation in our understanding of its role in pathogenesis of CAD and development of the potential therapeutic agents which might modulate its activity, expression in circulation and tissues. In the present review, we discuss the structure and activity of human PON3 enzyme and its Single nucleotide variants that could potentially lead to new clinical strategies in prevention and treatment of CAD.

## 1. Introduction

Cardiovascular diseases (CVDs) are the foremost cause of worldwide mortality, including India, and by 2020 coronary artery disease (CAD) is expected to become the largest contributor to this growing burden [1,2].

Ethnic and regional variations prevail for risk factors of developing CVD. Asian Indians are three to four times more susceptible of developing CVD than Caucasians, six times more than Chinese, and 20 times higher than Japanese [3,4]. Moreover, Indians are prone to develop CAD at a younger age [5,6]. Atherosclerosis is considered to be the fundamental pathogenetic mechanism for causation of multi-factorial CAD [7]. The common modifiable risk factors are dyslipidemia, hypertension, smoking, and sedentary lifestyle whereas non-modifiable risk factors are age, gender, and genetics [8]. For development of atherosclerosis, low density lipoprotein-cholesterol (LDL-C) is the major target for oxidation as oxidized LDL-C is involved in both its initiation as well asin progression [9,10].

Research over time has shown a definite role of genetic factors in susceptibility to CAD and significant advances have been made in identifying potential candidate genes which predispose individuals towards the disease. These genes have been identified by different strategies such as linkage studies, association studies with candidate genes, and whole genome wide association [11]. Epidemiology demonstrates an inverse relationship between CAD risk and high-density lipoprotein-cholesterol (HDL-C) concentration [12,13]. Studies governing preventive mechanism of CAD have shown that HDL-C exhibits its antioxidant role due to its association with a crucial esterase i.e., paraoxonase [14] besides platelet activating factor acetyl hydrolase [15] and lecithin cholesterol acyl transferase [16]. Paraoxonases are a family of three enzymes, with lipolactonase activity, that degrade lipoperoxides in lipoproteins and cells. These three enzymes share overlapping properties with some differences and gene encoding these enzymes harbors polymorphisms which mask their catalytic activity [14,17]. In context of population genetics, the measurement of Paraoxonase activity and concentration (PON status) are considered to be more important than studying *PON* polymorphisms alone [17] when the role of these genes is dissected to determine any association with the progression of disease. Studies are lacking in various ethnic groups who have different *PON* polymorphisms [18]. The present review discusses the available information on less investigated PON3 status i.e., activity and concentration and its genetic polymorphisms and their possible role in CAD.

## 2. Paraoxonases (PONs)

Remarkable evidence has been produced in the past two decades about the role of paraoxonases in atherosclerosis. PON1 (E.C. 3.1.8.1) was the first identified paraoxonase to play a role in CAD. It hydrolyzes diazinon, chlorpyrifos (an oxon metabolite) and nerve gases [19] (e.g., sarin and soman). It is an HDL-associated enzyme synthesized mainly in the liver and prevents LDL-C from oxidative modification [20].

## 3. Paraoxonase Gene Cluster

It comprises *PON1, PON2,* and *PON3* genes positioned on chromosome 7 (q21, 22) in humans and on 6 in mouse between q22.3 and q23.1 [21], approximately 27–28 kb [22,23]. These genes may have derived from the common precursor and share structural homology and 70% nucleotide identity [22]. An extra nucleotide residue in exon 4 of *PON1* codes for 105th amino acid but is absent in *PON2* and *PON3* which makes it unique in function. A gene coding for one of the pyruvate dehydrogenase kinases (*PDK*) [21,24] is located next to the *PON* cluster, and is sufficient to link the PON1 genotypes with diabetic glycemic control in several studies [14].

## 4. Evolution of *PON* Genes

The evolutionary origins and substrate specificities of PONs is poorly understood. Experiments in the early 1990s, used bacterial systems using *Escherichia coli* [25,26], yeast using the *Pichia pastoris* expression system [27], and in insect cells using Baculovirus assemblies [28] to express and purify PON1,PON2 and PON3 proteins and Zhu et al. in 2006 used silk worms for the same purpose [29]. A large body of literature presents traces of PONs from primitive prokaryotes to present day evolved human race. The *PON3* gene is conserved in mammals, chimpanzees, Rhesus monkeys, dogs, cows, mice, rats, *Caenorhabditis elegans*, and higher vertebrates. Only one Paraoxonase gene is present in birds, frogs, and fish, having the highest percentage identity with *PON2*. However, its homologues are known in the worm *C. elegans*, plants, bacteria, and fungi [15,22]. Bacterial systems do have a presence of *PON* genes. However, not all bacteria carry *PON* genes, the exception is mostly Gram negative ones in which acyl homoserine lactones (AHLs)as quorum sensing signal molecules are abundant e.g., *Pseudomonas aeroginosa, Vibrio fisheri*, etc. [30,31,32,33]. To these AHLs, mammalian PONs act as quorum quenching and degrade the biofilms created by bacteria. *PON* like genes, namely “Partner of Numb”, 672 amino acid long, 72KDa in other model organisms like *Drosophila melanogaster* (representing insects) play a role in embryonic tube development and asymmetric neuroblast division and its predominant expression is reserved in embryos [34]. In addition, in higher vertebrates (birds, amphibia, reptiles) *PON* like genes are present but no *PON* genes have been identified in viruses till date. In the case of protozoa and primitive metazoan, the PONs are likely to relate to innate immunity rather than diverging to detoxification functions which could have been the result of a gene duplication event [35]. Such genes do also exist in the genomes of extinct human subspecies (*Homo neanderthalensis*, *Homo sapiens denisovan*) and other primates when *insilico* search tools for comparison with the human situation were done on the NCBI database for PONs (using key terms PON3, *Homo sapiens*). For a more detailed analysis of the distribution of paraoxonase genes in the three kingdoms of terrestrial life extensive literature has yet to be evaluated to discuss the detailed origin and function of each of these enzymes. A total of 114 organisms have orthologs with human PON genes, sequences of which are available on NCBI database.

Interestingly, the first organophosphate-degrading bacterial species, *Flavobacterium* sp. ATCC27551 possessed an *opd* gene that showed transposon-like architecture, with widespread distribution of the gene among other microbial species which hydrolyze organophosphates(OPs) containing the *opd* (harboured by fungi, cyanobacteria, E. coli (*yhfV*) andMycobacterium tuberculosis) which might have occurred through lateral DNA transfer [36,37]. These hidden facts connect evolution of PON enzymes from primitive prokaryotes to a higher order of hierarchy in mammals and then to humans. According to phylogenetic analysis, PON2 is the eldest and PON1 is the newest of the PON family [15]. PON1 and PON3 reside in the cholesterol-carrying particle HDL, whereas PON2 is found in tissues [16] and all are calcium dependent [25,38]. Unlike human PON1 and PON3, PON2 is intracellular being coupled with membrane fractions [19,22], found in the endothelial cells, smooth muscle cells, and macrophages of the arterial wall and is absent in HDL, LDL, or the media of cultured cells [22].

## 5. Paraoxonase 3 (PON3) 3.1.1.2

Credentials to human *PON3* gene identification were documented first from the genome data base (GDB) by Primo-Parmo et al. [22] in 1996. It was similar but not identical to PON1. Ozols (1999) purified the same protein [39] from rabbit liver microsomes. After sequencing, it showed 60% identity with rabbit serum PON1 and 84% similarity with the PON3sequence as given by Primo-Parma et al., covering 350 residues with non-glycosylated hydrophobic amino terminus. Likewise, Draganov et al. [14] were the first topurify and characterize a mammalian (rabbit) plasma PON3. They also expressed the rabbit PON3 cDNA in 293T/17 cells and found the same specificity (to hydrolyze lactones and not OPs, a necessary step for its purification) and the same molecular mass of the cloned enzyme as that of the rabbit serum purified one. Later on, Rodrigo and his team worked on purification and characterization of rat liver microsomal PON3 [40] protein. It was 95% identical with the deduced cDNA sequence of the mouse PON3, 67% identical to rat PON1, and with similar homology between PON1′s and PON3′s of other species. Brushia et al. [28] then expressed and purified PON1Q192 from baculovirus assemblies. Subsequently, Draganov et al. [41] purified all the three human PONs from the same expression systems. Then expression of active human PON3 protein appeared as inclusion bodies in *E. coli* by Lu et al. [25]. It was soluble with high levels of Triton X-100.

*PON3* is sited between *PON1* and *PON2* and is the least studied compared to other *PON* homologues. In both human and mouse all three PONs contain nine exons of approximately the same length, eight introns, and TATA-less promoters [22]. *PON1* has 355 amino acid residues whereas *PON2* and *PON3* have 354 amino acids. At codon position 106, lysine in human *PON2* and *PON3* is missing [22,42]. All the amino acids required for PON3 activity are conserved between the human, rabbit, and mouse sequences [40,43]. These residues are also preserved in *PON1* and *PON2*. The conserved histidine at position 243 and tryptophan at position 281 in both *PON1* and *PON2* are replaced by lysine and leucine respectively in *PON3* of both human and murine sequences [44].

## 6. PON3: Single Nucleotides Variants (SNPs) and Haplotypes

Studies in the 1960s and 1970s demonstrated polymorphic distribution of PON1 activity and frequency of respective phenotypes among populations of different ethnic groups [45]. The *PON2* and *PON3* knowledge was extremely limited in the late 1990s, although few emerging studies reported genetic associations with metabolic diseases [46,47]. The SNPs of paraoxonases, mainly *PON1*, were reported in literature later on, especially their significant role in CAD causation. Furthermore, experimental findings quoted antioxidant behavior of PON2 and PON3 but were silent regarding involvement of their respective SNPs. Presently, information about *PON3* genotypes and its SNPs is scarce [48,49] and the influence of such substitutions or deletions is unknown in various intermediary pathways where enzyme levels are of clinical significance. A prospective Northwick Park Heart study by Robertson et al. in 2003 reported twoSNPs of h*PON3* genes at positions 99 alanine (GC**G**) to alanine (GC**A**) and at 107aspartic acid (GAC) to asparagine (AAC). However, these SNPs have not revealed much information about its association with CAD [50].

Whereas, Campo et al., when compared their data to *PON1* and *PON2*, no polymorphism could be identified for *PON3*. However, serine to threonine substitution around position 311 and glycine to aspartic acid at 324 position were detected in a southern Italy population [48]. The functional consequences, however, of these polymorphisms regarding CAD development is not clear. The first coverage report governing the alliance of the *PON3* gene with serum PON1 activity in systemic lupus erythematosus (SLE) and atherosclerosis risk, did not reveal any significant association with PON3 variants [51]. Interestingly, two SNPs had significant influence on PON1(phenyacetate) activity, out of total six studied variants and accounted for 3% of variation in the PON1 activity, emphasizing neglected variants to be identified. In spite of *PON3* promoter gene sequencing or numerous case-control studies aiming to determine ability of the variants to protect LDL from oxidation, rarely any of the variants had demonstrated an influence over gene expression to date except in one or two studies [50,52], with no conclusive results about *PON3* association with CAD risk as summarized in the Table 1.

Often, these might have included inappropriate sample size, population heterogeneity, and/or used different genotyping approaches that are difficult to interpret. Although, huge inter-individual variation in PON3 activity/concentration has probably contributed to some extend defense against CAD. Notably, Wang et al. detected −133 C >A polymorphism in the *PON3* gene [53] which was located at a potential binding site for transcription factor LFA-1(Integrin Lymphocyte Function- associated Antigen) but no report to date is available regarding role of LFA-1 in PON3 activity and expression regulation, although LF-Al has its interaction (binding sites) with the promoter regions of several liver-specific genes like *APOA1, APOB1, APOA4*, and pyruvate kinase [54]. LFA-1, a receptor for intercellular adhesion molecules (ICAMs), consists of integrin alpha L chain which combines with the beta-2 chain to form it. LFA-1 plays a key role in leukocyte intercellular adhesion through interaction with ICAMs, like HDL [55,56,57]. PON3 association with HDL and involvement in immune response [58] may pave the road to investigate a further role of immune regulation of HDL, and, specifically, PON3 activity attributed by its respective SNPs. *PON3* promoter characterization becomes necessary for understanding its gene regulation so as to elucidate its role in treatment.

Aragones et al. [52] demonstrated six functionally active promoter polymorphisms of the *PON3* (−567 C/T, −665 A/G, −746 C/T, −4105 G/A, −4970 T/G, −4984 A/G), with significantly moderate influence on PON3 circulating levels. Typically, the least frequent genotype showed 10% reduction in serum PON3 levels, compared to most frequent genotype indicating strong association and confirmed TGTAGG, TGTGTA, CACGTA being the most frequent haplotypes influencing serum PON3 concentration when adjusted for gender, age, and body mass index [56]. Another investigation in our laboratory also could not detect previously reported two SNPs (i.e., D107N and G324D) in our population groups, however, the polymorphism in coding region at A99A resulted in significantly low PON3 activity and low PON3 circulatory concentration in these individuals who harbored the A99Aheterozygous mutant in particular [59] (full text not published, for more details see Table 1). Therefore, current findings strongly emphasize the need to explore more information on *PON3* to get a better understanding of CAD as well as particular genotypes associated with it. As there is a paucity of studies on *PON3* polymorphisms amongst various population groups at present, it is worthwhile to undertake polymorphisms into account in a homogeneous population to find the genetic basis of disease. Besides these, it is also not known which alleles in the *PON3* have greater activity for its natural substrate in vivo.

## 7. Substrate Specificities and PON3 Status

All three PONs metabolize derivatives of arachidonic acid (5-hydroxy cicosatetraeomic acid 1,5 lactone and 4-hydroxy docosahexaenoic acid) whereas PON3 is exclusive to lactones hydrolysis such as lipid-lowering statin pro-drugs lovastatin and simvastatin [60] and the diuretic spironolactone, canrenone, hence exhibits lactonase activity. Organophosphates are exclusively hydrolyzed by PON1 whereas PON3 possesses the most limited arylesterase activity and is devoid of organophosphatase activity against the synthetic substrates like paraoxon and phenylacetate [14]. Aceylhomoserine lactones are hydrolyzed by paraoxonases, and are now accepted as chief quorum sensing indicators in bacterial virulence, and modulators of host anti-inflammatory response [30,32]. To answer, how PON3 imparts anti-atherogenic activity and its endogenous substrates remains largely unknown despite its usual task as paraoxonase/arylesterase. Moreover, laboratory evolution [31] and structural activity studies on PON1 [61] indicate that PON1 was the first detected lactonase among PON members [62]. Now lactonase activity is the characteristic feature of all PONs. All PONs have similar active site residues resembling one another, but PON1 variants have evolved with different activity patterns [31,63] with due course of evolution. Thus, derived lactones from fatty acid oxidation may constitute the native substrates of PON1 [41,64].

Fatty acid esters, fatty acid ethers whether exogenous or endogenous (such as Arylesters, HTL) and cyclic carbonates are only hydrolyzed by PONs [31,42,61] whereas peptides, oligonucleotides, oligosaccharides, or epoxides are not hydrolyzed by Paraoxonase isoforms. Following the discovery of PON2 and PON3, these were also termed paraoxonases although neither one hydrolyzed Paraoxon [22]. After discussions on the appropriate nomenclature for the PONs, naming of the three PONs should be delayed until the natural physiological substrates are identified [42] which remains an open question to date and searching for its alternative substrates and catalytic activity in different species across prokaryotic bacteria to mammalian cells is in progress to achieve alternative functions harbored by these three enzymes. Previous structure-activity relationship studies demonstrate that PONs have a high specificity for lipophilic lactones, suggesting that such compounds may be representative of native substrates. This report describes the ability of PONs to hydrolyze two bioactive d-lactones derived from arachidonic acid, 5,6-dihydroxy-eicosatrienoic acid lactone (5,6-DHTL), and cyclo-epoxycyclopentenone (cyclo-EC). Both lactones were very efficiently hydrolyzed by purified PON3. PON1 efficiently hydrolyzed 5,6-DHTL, but with a specific activity about 15-fold lower than PON3. Studies with the PON inhibitor EDTA and a serine esterase inhibitor indicated that the PONs are the main contributors to hydrolysis of the lactones in human and mouse liver homogenates [65].

## 8. Amino Acid Sequence Similarity

When the amino acid sequences of the known paraoxonase isoforms are compared with other known hydrolyzing enzymes, hardly any structural similarities do occur. Comparison of serum acetyl hydrolase (PAF-AH) and paraoxonase 1 (PON1) has been already evaluated by ErdalBenli et al. [66]. Additionally, Russell L. Carr et al. [67], found species differences in paraoxonase mediated hydrolysis of severalorganophosphorus insecticide metabolites. These differences are possibly due to effects on either posttranslational modification of the catalytic structure of PON1 or the induction of other factors that play roles in the activity of PON1.

Bacterial phosphotriesterases (PTEs), amidohydrolase superfamily, differ from the eukaryotic organophosphatases; PTEs are (β/α)_8_ barrels with an active site containing two transition metal ions such as Co^2+^, Mn^2+^, or Zn^2+^. PTE from *Pseudomonas diminuta* hydrolyzes paraoxon, Dihydro-caumarin(DHC), and other lactones whereas bacterial lactonases, dubbed PTE-like lactonases (or PLL), have been identified to possess both lactonase and organophosphatase activities [38]. Lactones are natural compounds, many of them with high biological activity, while organophosphates are human-made chemicals introduced in the 20th century [38]. Thus, it is plausible that lactonase is the primary activity for which the enzymes discussed here evolved for, while the organophosphatase activity arose as a promiscuous activity during their evolution.

## 9. Paraoxonase3 Structure: (PDB 1v04)

The crystal structure of serum paraoxonase 1 has been deduced [31,32,38,61,62,63]. However, research on more catalytic functions focusing PON3 is not yet evaluated. Many functions of this enzyme and why differences in PON3 and PON1 occur are still not clear. All PONs utilize a catalytic calcium ion, which functions as an oxy-anionto stabilize substrate and reaction states. Additionally, this enzyme active site employs two histidine residues (His115 and 134) involved in proton transfers, a glutamic acid(Glu53) to stabilize reactive hydrogens, and an asparagine(Asn168) to stabilize transition states and intermediates in the active site [31,38,62,63,68]. The exact mechanism is still a subject of further research and it is suggested that the His115 residue is not necessary for the lactonase and arylesterase activity of the enzyme [69].

The enzyme status of PON3 which encompasses its activity and circulatory concentration needs to be quantified as it essentially upholds the predetermined task in disease development or progression [70]. Despite similarity in residues, the active site of PON3 is larger than that of PON2 and PON1. Thus, it has greater ability to hold bulky statin lactones and spironolactone, whereas PON1 is capable of hydrolyzing only non-substituted and short chain-substituted lactones [38]. The physiological substrates of PON3 are still not known and are under investigation, which may better help to determine functional aspects of PON3 as a therapeutic candidate. There is no direct measurement for PON3 activity as information about its exact physiological substrates are uncertain and statinase activity is exclusively known to it [60]. Various researchers have performed DHC based [14,40,71] and statin based estimation [60] to indirectly correlate PON3 activity from serum and have reported its lower levels in obese as compared to slim school children [60] keeping in mind its more specific and exclusive substrates. This sheds light on new avenues in diagnosis, especially as an early indicator of susceptibility to arteriosclerosis as well as an indicator of potential drug side effects.

## 10. PON3 Concentration

The efficiency of PON3 to prevent copper-induced LDL oxidation is 100 times more than PON1 but is found about 200 times less abundantly than PON1 in rabbit HDL [14]. In mice, PON levels are negatively correlated with atherosclerosis [72,73,74]. Indeed, PON3 is atheroprotective, but a dearth of reliable methods to estimate its concentration in the serum is one of the hurdle which prevents its use for therapeutic benefit. Aragones et al. [52] developed enzyme-linked immunosorbent assay (ELISA) to measure serum PON3 concentration in a population-based study. They found its concentration decreases with growing old. However, no significant association could be drawn with sex, body mass index, or smoking status [52]. Thus, PON3 serum concentration and activity are to be predetermined when carrying out association studies. Therefore, PONs give atheroprotection by two means, firstly, by exerting their effect on HDL particles(PON1 and PON3) and secondly being friendly, intracellular PON2 protects macrophages and possibly other cells of arterial wall [75].

## 11. Histological Distribution

PON3 is produced primarily in the liver and released into the blood circulation, where it gets bound to HDL and apolipoprotein A1. IHC (Immuno-histological-chemistry) sections from different tissues show its variable distribution in body, whereas *PON3* gene expression is chiefly observed in the liver and kidney [76]. The defensive nature of PON3 probably goes beyond circulation, since it is markedly distributed to various tissues including the lung, liver, pancreas and intestine, and eye [77]. Thus, immuno-histochemical localization can provide a better platform to investigate the changes in the distribution of these enzymes in a variety of diseases, stage specific changes in cell-subtypes, and it can be correlated with various pathways involved in pathological condition if its subcellular localization becomes clearer.

## 12. Antioxidant Potential

Liver is the primary site of lipid metabolism [78]. Ox-LDL increases the generation of oxidative stress by increasing the production of intracellular ROS and lipid peroxidation products [10]. PON3 reduces early oxidative products and thereby suppresses the propagation of oxidation by hydrolyzing biologically active oxidized phospholipids and lipid hydroperoxides in oxidized LDL respectively [19,79].

Varied cellular (macrophages and Hepatoma cells) and experimental rodent systems (high fat diet mice, *apo E^−/−^* mice) illustrate oxidative stress has different influences on PON protein expression. Oxidative stress downregulates PON1, upregulates PON2, and unalters PON3 expression [80,81]. Evidently, cell culture study on mouse peritoneal macrophages (MPMs) from control and *apo E^−/−^* (rich in lipid peroxides) mice, incubated under an oxidizing environment (in the presence of oxidized phospholipids and supplied with high fat atherogenic diet) showed *PON2* mRNA expression and 50% elevated lactonase activity towards dihydro-coumarin, but unaffected *PON3* mRNA expression. Further, incubation with purified PON2 and PON3 demonstrated low cellular lipid peroxide content [82] in *apoE^−/−^* macrophages. This evidence needs more evaluation to draw out the exact relationship between macrophage oxidative stress, paraoxonase proteins, and atherosclerosis.

Dragnov et al. have reported the efficiency of purified rabbit PON3 in protecting LDL-C against oxidation is 100 times more than rabbit PON1 [83], besides its capability of preventing copper induced LDL-C oxidation in vitro [14]. Reddy et al. have shown that LDL-C when incubated with stably transfected cells over-expressing human PON3, have significantly less lipid hydroxides and poor monocyte chemotactic activity [80]. In addition to these, *PON3* has been linked to oxidative stress for the first time in the context of modulating the levels of reactive oxygen species (ROS) in ex vivo and in in vivo experimental models [84,85]. In association with Q10, it protects against mitochondrial oxidative stress [85]. Thus, it is important to carry out linkage studies in individuals of different genetic make-up with diverse ethnicities to discover its anti-atherogenic circuitry influencing the lipoprotein metabolism and mediating its role in mitochondrial function as well.

## 13. PON3 Regulation in Various Diseases

Regarding aforementioned antioxidant functions of *PON3*, it is difficult to state its regulation by inflammation and oxidative stress in atherosclerosis in the absence of its known physiological substrates. *PON3* appears to be influenced by oxidative stress. However, a few reports on knockout mice models suggest PON3 expression remains unchanged in response to oxidative stress. Shamir et al. reported Caco-2 cells subjected to Fe/Asc-induced oxidative stress showed no change in PON3 expression profile at gene and protein level with little reduction in statinase activity [86]. In a similar manner, *apo E^−/^^−^* MPMs, under stress conditions showed no significant change in PON3 mRNA levels even in the absence of statinase activity, but pomegranate juice/vitamin E supplementation augmented statinase activity in *apo E^−/^^−^* mice, indicating its regulation by oxidative stress [71,86,87,88]. In contrast, J774 A.1 murine macrophages-like cells showed reduced PON3 lovastatinase activity when pomegranate juice was given to these cells [89]. Pro-oxidative environment however reduced PON3 activity in MPM isolates from C57BL/6 mice without having any impact on gene expression [81]. Furthermore, *PON3* directed hepatic expression in murine model C57Bl6/J was not affected by genetic background or diet [90].

Further, its upregulated expression was reported in late gestation in fetal rat, sheep, and humans, which may prove it as an anti-oxidative entity in premature or newborn babies [91]. Additionally, obtained colonic samples from healthy individuals, patients of Crohn’s disease and ulcerative colitis by Boehm et al. [92] detected its mRNA in 1/14 cases with active ulcerative colitis and in 3/17 Crohn’s disease patients, compared to 10/25 controls [70], suggesting that inflammation and oxidative stress in inflammatory bowel disease patients may have downregulated intestinal PON3 expression which is similar to the results reported earlier in systemic/local inflammatory disoders e.g., ulcerative colitis, diabetic neuropathy and retinopathy, rheumatoid arthritis and glomerulonephritis, where serum Paraoxonase (PON1) levels and activity were found to be reduced [84,93,94]. On the contrary, its upregulated profile in a variety of tumor samples or in cell lines has also been documented [85]. Overall, these observations suggest the role of PON3 in atheroprotection via oxidative modification of lipids and in initiation of inflammatory events [95].

In support of this, Tward and Rozenberg et al. propose PONs involvement in vascular disease as its knock-out counterpart had shown increased susceptibility to atherosclerosis [96,97] and developed 42% excess in stenosis [98]. Similarly, the development of human *PON3* transgenic mice by Shih et al. [72], instruct better comprehension of atheroprotective actions of PON3, 4–7-fold more *PON3* expression in their liver, and presented reduced lesions comparable to control ones when given an atherogenic diet. Availability of knockout mice models for the different paraoxonase isoforms has now made it easier to understand more biological functions of *PON3* in different diseases. This has been facilitated by D.M. Shih and co-workers [99]. Notably, reduction in adiposity, atherosclerotic lesions, and monocyte chemoattractant protein-1-(MCP-1) in control and LDL receptor Knock Out (KO) mice overexpressing *hPON3* also suggests synergy of *PON3* in obesity control and atherosclerosis [72]. In the past, cholesterol transcriptional regulator, Sterol regulatory element binding protein-(*SREBP-2*),–104to –95bp triggered activity of*PON1* promoter in a dose-specific manner signified an extra lipid-associated mechanism of *PON1* activation [98]. These findings may pave our way of understanding a mechanistic approach towards another homolog of the *PON* family i.e., *PON3* and its role in prevention of coronary artery disease. Unfortunately, no report to date has accounted for LFA-1 transcription factor in *PON3* gene regulation despite LFA-l interaction with the promoter regions of several liver-specific genes [54]. Wang et al. detected −133 C>A polymorphism in the *PON3* [53] which was located at a potential binding site for LFA-1. Our team also made an attempt to study the functional consequence of this −133 C>A polymorphism in North West Punjabi Indians and we could only detect the polymorphic site but no significant association with CAD (Table 1). In view of poor understanding of PON3 with conflicting results, additional studies explaining its promoter interaction with the transcription factors can elucidate its regulation.

## 14. *PON3* Gene Expression

For further characterization of Human PON3 activity against atherosclerosis, gene expression studies have been performed which revealed human PON3 appears in widely distributed tissues [77]. However, its mRNA expression is precisely found in the liver and kidneys [76] and has been already discovered in murine macrophages with specific statinase activity [81]. Likewise, another genetically modified animal model over expressing *hPON3* with *apoE*^−/−^ KO background showed poor susceptibility of LDL to oxidation, as HDL had efficient efflux cholesterol from macrophages [90]. This study also established that mice HDL particles do not house PON3. Similarly, with administration of adenovirus expressing *hPON3*, reduced arterial lesion size was observed with no anti-inflammatory/antioxidative effects [90,100]. However, the anti-inflammatory potential was shown by a carbon-tetrachloride (CCl_4_) induced liver injury murine model over expressing *PON3* which led to increased glutathione levels whereas decreased malonyl-di-aldehyde (MDA) levels, tumor necrosis factor (TNF-α), and interleukin-(IL-1β), respectively [101,102]. Overall, these studies sustain *PON3* against atherosclerosis and antioxidant synergistically to be explored for future therapeutics besides its anti-inflammatory function.

## 15. Role of Environmental Factors

Risk factors associated with CAD are age, nutrition, lifestyle, smoking status, and alcohol consumption. PON3 activity decreases with age [52]. In elderly patients, there is an increased susceptibility of HDL to oxidation. Recent experimental findings have proven *PON3* involvement not only with CVDs, but with obesity also [72]. In general, sedentary lifestyle is equally associated with greater risk of CHD, stroke, hypertension, hypercholesterolemia, whereas regular physical activity improves quality of life and guards against the incidence and progression of CHD [103,104]. Effects of moderate aerobic training performed on a rat model by Rita et al. [89] confirmed first the upregulation of *PON3* mRNA, protein levels, and enzymatic activity, rather than *PON1*. Feasibility of knockout mice models for the different paraoxonase isoforms has made investigation of such animals easier, for understanding of more biological functions in different diseases also. These credentials have been supplemented by a group led by Shih et al. (2015) [99]. Transgenic male mice over-expressing *hPON3* generated by Shih et al. [72] also demonstrated elevated expression of *PON3* bestowed protection against obesity and atherosclerosis in these mice. These parallel results emphasize *PON3* as an attractive candidate for future studies in relation to its joint participation in CVD and obesity. Thus, sustained physical activity awards beneficial effects on lipoprotein metabolism, by decreasing plasma triglyceride and increasing HDL-cholesterol levels, which bears antioxidant properties and cardioprotection.

For centuries, one of the generally accepted notionss is that consuming fruits and vegetables and wine is beneficial for protection against heart disease. Polyphenolic compounds such as flavonoids quercetin and myricetin in these foods are responsible for their atheroprotective effect [105,106,107] and inhibit LDL oxidation in humans as well as in animal models [107,108]. On the other hand, tobacco use is strongly related to CAD. Smoking cigarettes or beedi is associated with increase in the risk of myocardial infarction [109]. Red wine is a rich dietary source for polyphenols. The phytoalexin in resveratrol [108] actively contributes to the beneficial effects of wine and also modulates PON1 expression [110]. Pharmacologically, significant micromolar units of resveratrol accumulation in liver [93] and in plasma have been reported after moderate wine intake. Therefore, pharmacological modulation of PON3 activity/gene expression might lead to a better therapeutic weapon against CAD and merits further research.

## 16. Summary and Conclusions

**Figure 1:**
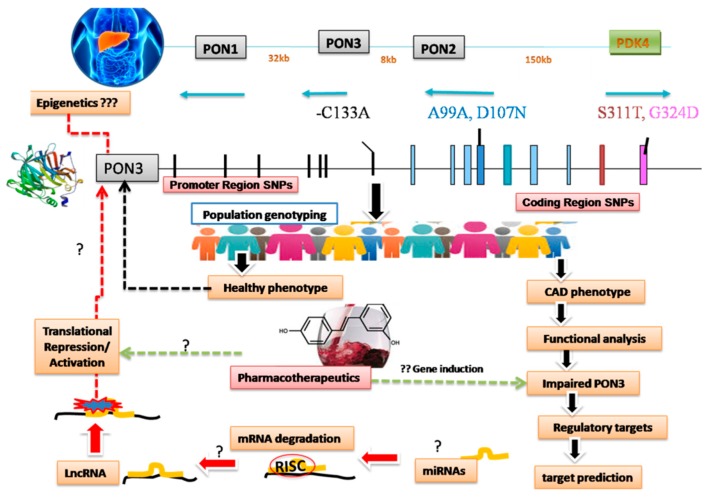
Schematic representation of interconnected hidden links to be explored in pathophysiology of coronary artery disease (CAD) associated with impaired Paraoxonases. sPON3 is synthesized in the liver and associated with high-density lipoprotein (HDL). PON gene cluster on chromosome 7 (q21, 22) comprises *PON1, PON2*, and *PON3* sharing homology with each other. Across the globe, several investigations in population genotyping have found very few polymorphic sites in the *PON3* (-133C/A in the promoter region and D107N, S311T, G324D in the coding region) which has a direct role in CAD progression except in one or two studies where serum levels and CAD phenotype have a positive correlation with the disease. Studies intending to report functional characterization of mutated *PON3* at regulatory level or epigenetic control could better predict the underlying mechanisms operating at transcriptional/translational levels. For this, miRNA moleculesmight give clues to identify its major targets as i.e., PON3. In this arm regulatory miRNAs and regulation pathways can be deduced in vitro or in vitro to better understand PON3 gene function and regulation so as to exploit the exact inducers of PON3 in translational pharmacotherapeutics.

The present review is an attempt to discuss the role of PON3 enzyme in CAD etio-pathogenesis beyond the thought of conventional Paraoxonases like PON1 (OP-hydrolyzing enzymes) which flock against cholinergic crisis. Paraoxonases act as crucial endogenous enzymes against oxidative stress which have been implicated in the pathogenesis of cardiovascular diseases. Emerging evidence supports the role of PON1 and PON3 (HDL associated enzyme) in prevention of atherosclerosis. It inhibits peroxidation ofLDL-C and inactivates LDL-derived oxidized phospholipids [14], thereby reducing its oxidized levels that are involved in initiation of atherosclerosis [10]. In vitro analysis of the recombinant HDL (rHDL) suggests that PON3 interacts with apoA1-HDL particles in a manner similar to that of PON1 [47,58]. This interface follows specific, inducible changes in the enzyme’s active site and increases stability of both rPON3- apoA1-HDL (half-life) in the presence of calcium chelating agent by >45-fold. Being antiatherogenic, rPON3 also stimulates the HDL-mediated cholesterol efflux from macrophages [47]. Its overexpression prevents atherogenesis in murine models [72,97,111]. However, in clinical settings, patients who represent fully established plaques, need care as it is too late for prophylactic approaches. Possibly all the three PON proteins play an important role in a number of inflammatory disorders. However, their exact mechanisms of action are not clear and need to be determined [112]. PON3, therefore, may be a potential preventive agent against atherosclerosis development provided identification of its lead substances, increasing or decreasing its endogenous levels [113] despite several explorations undertaken at gene/protein levels [48,50,52,114,115]. Downstream targets, epigenetic modifications, and regulatory microRNAs (miRNAs) can also be explored to identify the potential targets of *PON3* in addition to dietary interventions by polyphenols (Figure 1). Once regulatory circuits of *PON3* are deciphered, it would be easier to understand and translate these esterases for therapeutic benefit.

## 17. Future Perspectives

We are still lacking in paraoxonase research in terms of knowledge regarding understanding its pathobiology and designing therapeutics, despite its pivotal role in several diseases. Rare association studies, and lack of an accurate data regarding its physiological substrates, have limited our understanding of the pathogenesis of the disease. It is still unclear how these PONsdisplay a broad spectrum of activities if instructed/not by a single active site suggesting substrate specificities of these esterases could be under control of different genetic circuitry. There are now available knockout mice models for the different paraoxonase isoforms. It is worth investigating such animals for understanding of more biological functions in different diseases also. Thus, it becomes imperative to discover the potential candidates which modulate Paraoxonase 3 expression and help in improving its status.

## Figures and Tables

**Table 1:** Tabulated representation of studies, worldwide, deciphering *PON3* variants and investigating the role in coronary artery disease.

Study	Subjects Enrolled	*PON3* SNPs Studied	Outcome	Author, Year
Prospective Northwick Park Heart study II; to evaluate the effect of SNPs on CHD risk	3052 healthy men	A99A (GCG to GCA) D107N (GAC) to (AAC)	A99A SNP, did not revealed much information about its association with CAD but D107N was absent in the population.	Robertson et al. [50] (2003)
Polymorphisms screening of *PON* cluster in a Chinese Han population	949 subjects in the 474 cases, 475 controls	−133 C>A	Not found significant effect on CHD risk but detected -133 C >A SNP in *PON3* located at a potential binding site for transcription factor LFA-1(Integrin Lymphocyte Function-associated Antigen)	Wang et al. [53] (2003)
Identification of *PON3* mutations in a population of Southern Italy	1143 blood donors	G51G G73G A99A S311T G324D	G51G, G73G, A99A were silent and S311T, G324D were missense mutations with no clarity on function in CAD development	Campo et al. [48] (2004)
*PON* gene cluster TagSNPs analysis—on illumina platform	500 Caucasian males	*PON1*, *PON2* and *PON3*	No significant association with CAD disease	Carlson et al. [114] (2006)
Association study; *PON3* with serum PON1 activity, risk of atherosclerosis in SLE cases	377 cases and 482 controls (US whites and blacks)	*PON3* (A10340C, A2115T), *PON1* (L55M, Q192R)	All four SNPs explained 2%, 1%, 8%, and 19% of the variation in PON1 activity, respectively. *PON3* SNPs described only 3% of variation in PON1 activity	Sanghera et al. [51] (2008)
Influence of genetic polymorphisms of *PON* on lactonase activity	Healthy population	*PON3*−567, *PON3*−665, *PON3*−746, *PON3*−4105, *PON3*−4970, *PON3*−4984	Lactonase activity was lower than Paraoxonase activity which indicated evaluation of liver function in clinics	Marsillach et al. [115] (2009)
PON3; concentration determination and its association analysis with promoter polymorphisms	*n* = 356; 156 women, 200 men; mean age: 47 years, of Caucasian from the Mediterranean region of Catalonia	(-567 C/T, -665 A/G, -746 C/T, -4105 G/A, -4970 T/G, -4984 A/G)	Promoter SNPs associated with PON3 serum concentration. TGTAGG, TGTGTA, **CACGTA** haplotypes were significantly associated with changes in serum PON3 concentration when adjusted for gender, age, BMI	Aragones et al. [52] (2011)
PON1 and PON3; atorvastatin hydrolysis	Blood and liver tissues of patients undergoing surgery(*n* = 150)	-4984A/G, -4105G/A, -1091A/G, -746C/T and F21F	40 SNPs identified within the *PON*-locus associated with changes in atorvastatin δ-lactone hydrolysis and expression of PON1 but not PON3. Non genetic factors only were associated with PON3 expression	Riedmaier et al. [116] (2011)
Study of 51 common polymorphisms in the *PON* cluster	1328 Caucasian males	*PON3* (rs17884000, rs9640632, rs468, rs11768074 rs10487132 rs740264)	Predicted PON1 activity but not vascular disease	Daniel S. Kim et al. [117] (2012)
Case control study in North West Indian Punjabis	*n* = 300 cases, *n* = 300 proven CAD patients	C-133A, A99A, D107N, G324D	Low Paraoxonase 3 activity, circulatory concentration and A99A variants were predictive risks for angiographically proven CAD	K. Priyanka et al. [59] (2017)
